# A clinical and demographic analysis of oral pemphigus vulgaris: A retrospective cross‐sectional study from 2001 to 2021

**DOI:** 10.1002/hsr2.832

**Published:** 2022-09-13

**Authors:** Muhanad L. Alshami, Fawaz Aswad, Bashar Abdullah

**Affiliations:** ^1^ Department of Dentistry Dijlah University College Baghdad Iraq; ^2^ Department of Oral Diagnosis University of Baghdad Baghdad Iraq

**Keywords:** epidemiological, oral pemphigus vulgaris, prognosis, retrospective study, vesiculobullous

## Abstract

**Background and Aims:**

Pemphigus vulgaris is an autoimmune vesiculobullous mucocutaneous disorder with life‐threatening consequences. Early detection and adequate care are crucial for a good prognosis. This study aimed to determine the demographic data, clinical features, and the prognosis of patients with oral pemphigus vulgaris.

**Materials and Methods:**

From 2001 to 2021, all diagnosed oral pemphigus vulgaris cases were extracted. Each patient's demographic and clinical data were gathered. Patients were called via phone to assess the prognosis, treatment type, and specialty of the physician who provided the diagnosis and therapy.

**Results:**

The majority of the patients had only oral lesions with higher prevalence in female who also expressed severe pain than male. Only 14 of 29 patients responded phone calls. Except for one, all patients were in active disease. More than half of those respondents said pemphigus negatively affects social behavior and food intake. Correct diagnosis and treatment were decided by dermatology, oral medicine, and maxillofacial surgery specialists.

**Conclusion:**

Oral pemphigus vulgaris was prevalent in females. Severe pain was common in females and older people. Even with effective therapy, the prognosis was poor. Medical and dental professionals had little knowledge of pemphigus vulgaris. Patients frequently report poor quality of life.

## 1 INTRODUCTION

Pemphigus vulgaris (PV) is an autoimmune disease characterized by the formation of mucocutaneous blisters that soon ulcerate.^1^ The main pathogenic event is the production of autoantibodies against cellular adhesion molecules responsible for maintaining the integrity of the epithelial layers.^2^ Some of these proteins are more involved than others with PV, such as desmoglein3 (DSG3) in the mucous membrane (m) type of PV, while desmoglein1 DSG1 is known to be highly affected in the mucocutaneous (mc) type of PV.^3^, ^4^ The PV destructive process is briefly caused by antibody‐antigen interaction, resulting in the separation of epithelial cells and the formation of clefts just above the basal cell layer; these changes are the classic picture of PV under the microscope.^5^ People who have PV are more likely to get ulcers and erosive lesions because blisters in the mucosa or skin are fragile and have a high tendency to rupture.^6^

Although the incidence of PV is rare, ranging from 5 to 32 per 1,000,000 people/year with uneven geographic distribution.^7^, ^8^ However, unfortunately, the delayed diagnosis and improper management of PV would expose the patient's life to risk.^9^ Ethnicity may play a key role in PV development, for example, Jewish and Iranian people have a high PV incidence.^10^ Further, sex is another possible contributing factor. Some studies have indicated that PV is more prevalent in females than in males, whereas other epidemiological studies have shown no difference between both sexes.^11^, ^12^, ^13^ The most commonly involved site is the oral mucosa, in which about 90% of PV patients suffer from blisters and ulcers in different areas of the oral cavity, such as the buccal mucosa, floor of the mouth, tongue, lip, and palate.^14^ Oral PV negatively impacts the quality of life, and the patients complain of mouth aches and impairment in food intake.^15^, ^16^ Following involvement of the oral mucosa, skin and other mucus membranes such as ocular, esophageal, and genital may be involved. However, in a number of cases, the oral lesions are the only clinical presentation.^17^, ^18^

The diagnosis of PV relies mainly on the clinical manifestations and taking a biopsy from normal‐looking tissue near the lesions. The biopsy is used for histopathological examination and to detect the presence of immunoglobulins in the tissue sample by a direct immunofluorescent assay.^19^ Generally, there is no specific protocol for PV treatment, though corticosteroids and adjunctive treatments are commonly prescribed.^1^ This study aimed to determine the association of demographic data and clinical features with oral PV, together with the prognosis of patients with oral PV.

## 2 MATERIALS AND METHODS

The present observational retrospective study was started from September 2021 to January 2022 in the Department of Oral and Maxillofacial Pathology, College of Dentistry, University of Baghdad. The approval from the Ethics Committee, College of Dentistry, University of Baghdad (Ref. 297, April 2021) was obtained before commencing the study.

The present study followed a described by previous studies.^20^, ^21^, ^22^ The archived reports for all diagnosed cases in the oral pathological laboratory of oral PV from 2001 to 2021 were retrieved and used in this study. From the medical reports of each patient with oral PV, the following data were extracted: year of diagnosis, sex, age, type of PV (m PV or mc PV), oral manifestations, and site of the biopsy. Based on the level of approval, the authors were permitted to contact the PV patients via their given phone numbers listed in the reports. The main goal of this communication was to assess the disease prognosis and know the type of treatment. In addition, further questions were asked about the specialization of the physician who was contacted first by each patient, when signs and symptoms were appeared together with the specialty of the doctor who made the correct diagnosis and treatment. All questions were asked with the native language. The answers were obtained after explaining the aims of the present study to every patient and a consent was taken.

### 2.1 Statistical analysis

The data were analyzed using SPSS software (version 11.5). Frequency, percent, mean, and standard deviation were used for descriptive statistics. Chi‐square test was used for determining the association between dependent variables (sex and age) with independent variables including pain, oral clinical presentation, site, and type of PV. Association of the same dependent variables with the effect of PV on the diet and social behavior was also determined. Effect size was determined using odds ratio (OR) at 90% confidence interval (CI). Significant level was set at *p* < 0.05.

## 3 RESULTS

A total of 29 people were diagnosed with oral PV. Females accounted for more than two‐thirds of all reported cases. The average age of the oral PV patients was 43.5 ± 11.5 years old. The cheek was the most involved site in the majority of biopsies, followed by the lip and the tongue. Twenty‐three individuals showed a lesion that was only presented in the oral cavity at the time of diagnosis, while only six patients presented both with oral and cutaneous involvement. Other mucosal lesions (ocular and genital) were observed only in two patients. Oral PV was clinically manifested as ulcers in 69% of cases and as erosions in 31% of cases. Most of the patients had pain which was severe in most of the cases (Table 1).

**Table 1:** The demographic and clinical characteristics of oral pemphigus vulgaris (PV) patients

*Sex*		
Male^a^	6	20.7%
Female^a^	23	79.3%
Male: female	1:2.6	
*Age (years)*		
Mean ± SD	43.5 ± 11.5	‐
Min–max	23, 27–75	‐
*Site of oral biopsy* a		
Cheek	25	86.2%
Lip	3	10.3%
Tongue	1	3.4%
*Type of PV* a		
Mucus	23	79.3%
Mucocutaneous	6	20.7%
*Pain* ^a^		
Mild	5	17.2%
Moderate	8	27.6%
Severe	16	55.2%
*Clinical presentation* a		
Erosion	9	31%
Ulcer	20	69%

^a^ Frequency, percent.

**Figure 1:**
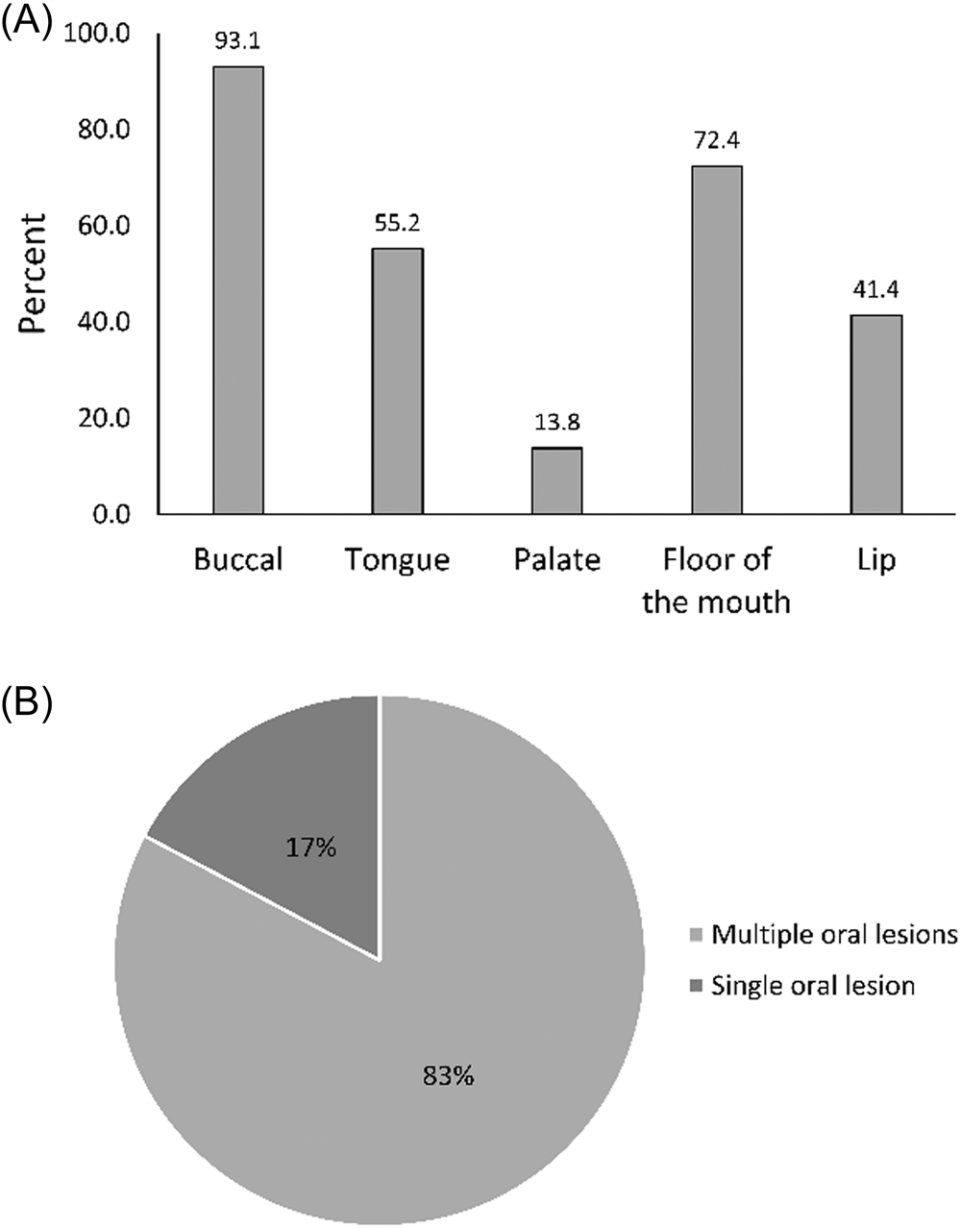
(A) The distribution of sites that are involved with oral pemphigus vulgaris (PV) lesions The most common site was the buccal mucosa (93.1%), followed by the floor of the mouth (72.4%), tongue (55.2%), and lip (41.4%). While the palate was the lowest site involved with the PV lesions (13.8%). (B) The distribution of patients according to the PV type at the diagnosis stage. Patients with m PV represented 83%, whereas patients with mc PV were observed in only 17% of the total cases.

More than 80% of the oral PV showed lesions within multiple areas of the oral cavity. The most common area was the buccal mucosa, followed by the floor of the mouth, whereas the palate was the lowest area involved with the PV lesions (Figure 1).

Statistically, a significant difference was found between both sexes in regard to the severity of the pain. Patients with oral PV (˃40 years) exhibited more severe pain than their younger peers (≤40 years). The clinical presentation, involved sites, and PV types showed no significant differences between males and females or between the age groups. However, m PV was shown to be associated with males more than mc PV (OR: 1.389) and patients aged ≤ 40 years (OR: 1.091) (Table 2).

**Table 2:** Association of age and sex correlation with other demographic variables

	Sex				Age (years)			
	Male^a^	Female^a^	OR	CI	≤40^a^	>40^a^	OR	CI^b^
Pain								
Mild	0, 0.0	5, 100.0	‐	‐	0, 0.0	5, 100.0	‐	‐
Moderate	4, 50.0	4, 50.0	0.000	0.000–0.957	6, 75.0	2, 25.0	0.000	0.000–0.368
Severe	2, 12.5	14, 87.5	0.000	0.000–4.000	9, 56.3	7, 43.7	0.000	0.000–0.523
*p*‐value*	**0.04**				**0.03**			
Oral clinical presentation								
Erosion	2, 22.2	7, 77.8	‐	‐	4, 44.4	5, 55.6	‐	‐
Ulcer	4, 20.0	16, 80.0	1.143	0.286–5.230	11, 55.0	9, 45.0	0.655	0.165–2.347
*p*‐value*	>0.99				0.70			
Sites								
Buccal mucosa	5, 18.5	22, 81.5	‐	‐	14, 51.9	13, 48.1	‐	‐
Tongue	3, 18.8	13, 81.2	0.985	0.287–2.982	10, 62.5	6, 37.5	0.646	0.216–1.753
Palate	0, 0.0	4, 100.0	‐	‐	2, 50.0	2, 50.0	1.077	0.239–4.819
Floor of the mouth	5, 23.8	16, 76.1	0.727	0.263–2.047	12, 57.1	9, 42.9	0.808	0.331–2.228
Lip	4, 33.3	8, 66.7	0.455	0.135–1.709	7, 58.3	5, 41.7	0.769	0.283–2.553
*p*‐value*	0.67				0.97			
Type of PV								
Mucus	5, 21.7	18, 78.3	‐	‐	12, 52.2	11, 47.8	‐	‐
Mucocutaneous	1, 16.7	5, 83.3	1.389	0.246–9.309	3, 50.0	3, 50.0	1.091	0.312–3.806
*p*‐value*	>0.99				>0.99			

Abbreviations: CI, confidence interval; OR, odds ratio; PV, pemphigus vulgaris.

^a^ Frequency, percent.

^b^ 90% CI.

* Bold font indicates significance at *p* < 0.05 by Chi‐square test.

The author responsible for communication was able only to contact 14 patients by phone (Table 3) including 11 women and 3 males, with an average age of 43.79 ± 13.92 years. Except for one patient who was cured after a year and a half of diagnosis and treatment, all other patients were in the active stage of the disease. The mean disease duration was 2.89 ± 2.66 years. All patients had m PV at the time of the initial diagnosis. Later, the type of PV converted to mc in 12 patients (85.7%). Corticosteroids and azathioprine were the drugs prescribed for all patients. Misdiagnosis was common among the first physician attached by the patients (*n* = 12, 85.7%). According to responses, the correct diagnosis and appropriate treatment were achieved by dermatology, maxillofacial surgery, and oral medicine specialists (Table 4). According to sex and age, 64.3% to 85.7% of the patients expressed a negative impact of PV on food intake and social behavior, respectively. The complaining from adverse social behavior was more in male (OR: 1.143), whereas the impact of the disease on food intake showed opposed pattern on male (OR: 0.200) (Table 5).

**Table 3:** Demographic and clinical variables according to the responses of pemphigus vulgaris patients

Variables	
Age (year)	43.79 ± 13.92^a^
Sex^b^	
Male	3, 21.4
Female	11, 78.6
Duration (years)	2.89 ± 2.66^b^
First presentation site^b^	
Mucus	13, 92.9
Cutaneous	1, 7.1
Another site involved later	
Yes	12, 85.7
No	2, 14.3
Healing	
Yes	1, 7.1
No	13, 92.9
Total	14, 100

^a^ Mean ± SD.

^b^ Frequency, percent.

**Table 4:** First attachment of the patient, primary diagnosis, correct diagnosis, and prescription of treatment according to the specialty of physician

Patient	First physician attached	Correct primary diagnosis	Correct final diagnosis	Prescription of treatment
#1	Otolaryngologist	‐	Dermatologist	Dermatologist
#2	Otolaryngologist	‐	Dermatologist	Dermatologist
#3	Nurse	‐	Maxillofacial surgery	Dermatologist
#4	General dentist	Yes	Oral medicine	Dermatologist
#5	General medicine	‐	Maxillofacial surgery	Dermatologist
#6	General dentist	‐	Maxillofacial surgery	Dermatologist
#7	Otolaryngologist	‐	Dermatologist	Dermatologist
#8	General medicine	‐	Maxillofacial surgery	Dermatologist
#9	General medicine	‐	Maxillofacial surgery	Dermatologist
#10	Otolaryngologist	‐	Oral medicine	Dermatologist
#11	General medicine	‐	Oral medicine	Dermatologist
#11	General medicine	‐	Oral medicine	Dermatologist
#12	Dermatologist	Yes	Dermatologist	Dermatologist
#13	General dentist	‐	Dermatologist	Dermatologist
#14	Otolaryngologist	‐	Dermatologist	Dermatologist
	14, 100^a^	2, 14.3^a^	14, 100^a^	14, 100^a^

^a^ Frequency, percent.

**Table 5:** Age and sex impaction on the social behavior and food intake

	Male^a^	Female^a^	OR	CI	≤43^a^	>43^a^	OR	CI
Effect on diet								
Yes	2, 16.7	10, 83.3	0.200	‐	6, 50.0	6, 50.0	1.000	‐
No	1, 50.0	1, 50.0		0.0211–2.590	1, 50.0	1, 50.0		0.098–10.210
*p*‐value*	0.40				>0.99			
Effect on social behavior								
Yes	2, 22.2	7, 77.8	1.143	‐	4, 44.4	5, 55.6	0.533	‐
No	1, 20.0	4, 80.0		0.173–9.589	3, 60.0	2, 40.0		0.115–3.271
*p*‐value*	>0.99				>0.99			

Abbreviations: CI, confidence interval; OR, odds ratio.

^a^ Frequency, percent.

* Significance at *p* < 0.05 by Chi‐square test.

## 4 DISCUSSION

PV is autoimmune blister/ulcer‐forming disease with a high fatality rate.^1^ The key findings of the current observational study were that females were affected more than males and the main age was 43 years. The first PV lesions were presented in majority of cases within the oral cavity. The sex (female) and aging were associated with increasing severity of the pain. Only 14 patients responded to the phone call and all of them were treated with corticosteroids and azathioprine with remission reported by only one patient. In general, a delay in intervention was observed in most of the patients.

Our study showed that females were more affected with PV than males. This outcome was in consistency with previous epidemiological studies worldwide.^24^, ^25^ The estrogen hormone, may play a major role in increasing PV incidence among females since this hormone has been reported to enhance immunological reactions.^26^ Other studies have suggested that estrogen may compromise the cellular adhesion apparatus.^23^ In contrast to our finding, some studies have indicated that male have higher PV incidence than females.^27^ The mean age for emergence of PV in the patients included in this study was the fourth decade. The results of other surveys conducted in different countries have reported a close result to ours.^11^, ^28^, ^29^ However, in other studies, the mean age of PV has been reported in the fifth decade of life.^13^, ^30^ The size of the sample, genetic predisposition, and other factors could be the main reasons for these discrepancies in results.

The oral presentations of PV lesions were ulcers and erosions in 69% and 31%, respectively. These patterns were similar to findings of Mark and his colleagues who found in their study that oral PV appears in the form of ulcers in most cases.^31^ PV blisters have a high tendency for bursting within 24 h leading to discontinuity in epithelial cell layers. Therefore, detecting an intact blister during clinical inspection is extremely rare, and all oral lesions are manifested either as ulcers or erosions.^32^ Oral PV lesions may be seen in any area within the oral cavity; however, buccal mucosa is the most commonly involved site than other sites^33^, ^34^ which also was the pattern observed in the present study.

Severe pain was the major symptom reported by the patients in this survey. This finding was similar to previous studies which showed that pain is one of the major symptoms of PV.^35^ The breached epithelial‐barrier results in exposing highly‐innervated underlying connective tissue causing an exaggerated response to the external stimuli. In addition, the severity of the pain was significantly higher in females than males. Previous reports have suggested that the male have a higher pain tolerance/threshold than females.^36^ Specific explanation for this difference in pain experience between both sexes is not clear yet. However, downregulation of the estrogen hormone in females with aging may be attributed to the increased pain sensation.^37^

The medical records reviewed in this study revealed that at the time of diagnosis, mc PV accounted for less than a quarter of cases, while the majority of individuals had m PV lesions, particularly the oral mucosa. Only two patients demonstrated lesions involving the eye and genital mucous membranes, in addition to the oral cavity. The reason for this variation in the clinical presentation could be explained on the basis of PV‐associated immunological response against antigens of cellular junctions. While in m PV autoantibodies are formed to DSG3, the prominent antigen expressed in the mucus membrane, mc PV is associated with autoantibodies to DSG3 and DSG1 which are expressed simultaneously in mucocutaneous regions such as skin.^38^

Only 14 (48%) out of 29 PV patients responded to the phone calls. Changing the phone number, traveling to other country, or the possible mortality of nonresponding patients may be the reasons that prevent communication with them. According to the responding patients, 80% of the cases were classified as m PV type which was switched later to mc PV type. This result was consistent with previous studies which showed that in most cases of PV where the initial lesions appeared first in the mucus membrane of oral cavity, while the skin lesions, if present, were either synchronized with the lesions of the oral cavity or appear several months later.^39^ More than 90% of the patients who were contacted still had PV lesions and were under treatment which included corticosteroids and azathioprine. The duration of disease from time of diagnosis ranged from 1 to 8 years, which is in line with the findings of previous studies.^13^ Interestingly, only one patient, who exhibited skin lesions first, reported remission 1.5 years after treatment. Studies have suggested that PV firstly manifested as oral lesions was denoted as a marker for poor prognosis.^40^, ^41^

The phone interview revealed that most cases were delayed and misdiagnosed by the first physician. This outcome was also documented in previous studies.^6^, ^42^ Dermatology, maxillofacial surgery, and oral medicine specialists were found to be highly responsible for correct diagnosis and management. Sex and age of patients with PV did not express differences in terms of social behavior and diet intake; however, indeed, the latter two domains were negatively affected by PV in the majority of cases. This coincides with other results that have to pinpoint the adverse impact of PV on the quality of life.^43^, ^44^

The absence of clinical examination and the need to a larger sample are the main limitations of the present study. In addition, prognosis was determined by interview via phone call rather than re‐examination clinically. Further, results of observational studies suggest association not causality which is determined by higher‐level clinical trials. However, the current study reported results of a rare disease associated with a high mortality and lowering quality of life but caution is advised for interpreting these findings to clinical practice until further confirmed by other studies.

## 5 CONCLUSION

Results suggested that PV was more prevalent in females. Severe pain in PV patients is highly associated with females and older age groups. Generally, the prognosis of PV was unfavorable even when proper treatment was administrated. Knowledge and awareness about PV were low among medical/dental personnel. Adding PV to the list of painful oral conditions is highly encouraged together with increasing the awareness of dental care providers about this disease via continuous education programs. In addition, Adverse impacts on the quality of life are common features in patients with PV.

## AUTHOR CONTRIBUTIONS

**Muhanad L. Alshami**: Data curation; Investigation; Methodology; Writing – original draft; Writing – review & editing. **Fawaz Aswad**: Methodology; Supervision; Writing – review & editing. **Bashar Abdullah**: Conceptualization; Supervision; Validation; Writing – review & editing. All authors have read and approved the final version of the manuscript. Muhanad L. Alshami had full access to all of the data in this study and takes complete responsibility for the integrity of the data and the accuracy of the data analysis.

## CONFLICT OF INTEREST

The authors declare no conflict of interest.

## ETHICS STATEMENT

Ethical approval for this study was obtained in accordance with declaration of Helsinki.

## TRANSPARENCY STATEMENT

The lead author Muhanad L. Alshami affirms that this manuscript is an honest, accurate, and transparent account of the study being reported; that no important aspects of the study have been omitted; and that any discrepancies from the study as planned (and, if relevant, registered) have been explained.

## Data Availability

The data that support the findings of this study are available from the corresponding author upon reasonable request.
